# Assay-guided treatment sequencing in chronic lymphocytic leukemia (CLL): a cost-effectiveness analysis

**DOI:** 10.1038/s41598-024-68431-6

**Published:** 2024-07-27

**Authors:** Samantha Pollard, Brandon Chan, Alina S. Gerrie, Adam J. N. Raymakers, Dean A. Regier

**Affiliations:** 1 Cancer Control Research, BC Cancer, Vancouver, Canada; 2 Faculty of Health Sciences, Simon Fraser University, Burnaby, Canada; 3 Division of Medical Oncology, Faculty of Medicine, University of British Columbia, Vancouver, Canada; 4 School of Population and Public Health, Faculty of Medicine, University of British Columbia, Vancouver, Canada; 5 BC Cancer, Centre for Lymphoid Cancer, Vancouver, Canada

**Keywords:** Haematological cancer, Cancer, Health care, Health care economics, Health policy, Public health

## Abstract

Costly targeted cancer treatments challenge publicly-funded healthcare systems seeking to align expected benefit with value for money. In 2021, The Canadian Agency for Drugs and Technologies in Health (CADTH) published a provisional funding algorithm for risk-based treatment of chronic lymphocytic leukemia (CLL). We estimate the cost-effectiveness of this algorithm against current standard of care. We constructed a probabilistic Markov model comparing next generation sequencing (NGS) assay-guided front-line treatment of acalabrutinib versus venetoclax with obinutuzumab to a comparator wherein patients initiate acalabrutinib. The primary outcome was the incremental cost-effectiveness ratio (ICER) per quality-adjusted life-year (QALY) gained. Analyses were conducted from the British Columbia healthcare system perspective, with outcomes discounted at 1.5%. Assay informed treatment for patients with CLL resulted in an incremental cost effectiveness ratio of $18,040 (95% CI $16,491–$19,501) per quality adjusted life-year (QALY) gained. The probability of the NGS guided treatment algorithm being cost effective was 80% at a willingness to pay threshold of $50,000 and a corresponding ICER of $18,040. Assay-guided treatment sequencing adds additional costs to healthcare but may be a cost-effective intervention for adult patients with CLL. Integration of real-world evidence would improve the validity and reliability of model estimated for decision-makers.

## Introduction

Chronic lymphocytic leukemia (CLL) is the most common leukemia in adults in Western countries, making up 25–35% of diagnoses^1^. The incidence of CLL is estimated to be 6.3 cases per 100,000 men and 3.3 cases per 100,000 women^2^. Patients with CLL have favourable survival compared with other cancers, with five-year survival estimates approximating 90%, and a median survival of approximately 10 years; however, there is significant variability in survival based on a number of clinical and disease related characteristics^3,4^. Treatment is reserved for patients with advanced stage or symptomatic disease^5,6^.

With an increased understanding of heterogeneous disease trajectories, there has been considerable effort to develop prognostic and predictive biomarkers to guide therapeutic sequencing decisions. These biomarkers are now included in a number of genomic testing strategies, many of which are not routinely available in Canada. For CLL patients requiring treatment, multiple options are available in Canada including: chemoimmunotherapy (such as fludarabine, cyclophosphamide, rituximab [FCR] and bendamustine and rituximab [BR]), and small molecule inhibitors such as BCL2 inhibitors (venetoclax), PI3-kinase inhibitors (idelalisib), as well as Bruton tyrosine kinase inhibitors (BTKIs) (ibrutinib and acalabrutinib). These small molecular inhibitors are often combined with anti-CD20 monoclonal antibodies, obinutuzumab or rituximab^7,8^.

Emerging research efforts investigating novel targeted CLL treatments have led to a shift away from traditional chemotherapy towards small molecule inhibitors. Currently, two such competing treatment strategies demonstrate efficacy and are included in standard care treatment of patients with CLL. Venetoclax, combined with an anti-CD20 monoclonal antibody, is a time-limited treatment approach with demonstrated efficacy in first-line and relapsed/refractory settings^9,10^. Recent phase III RCTs show that venetoclax-based treatments generate survival benefit in comparison with both bendamustine and chlorambucil based strategies^9,10^. Comparatively, BTKis such as acalabrutinib demonstrate progression-free survival (PFS) and overall survival (OS) benefit in both first-line and relapsed/refractory settings, as evidenced by recently published RCT and network meta-analyses^11–15^. In comparison to time-limited venetoclax-based therapy, BTKis are administered indefinitely or until disease progression, and are associated with high treatment costs^12,16–18^.

Currently, there is limited data to inform prioritization of BTKis versus venetoclax-based regimens in first-line therapy of CLL, challenging physician and reimbursement decision-making^7^. Further, no clear evidence exists to inform appropriate treatment sequencing for high-risk patients harbouring deletion 17p (*del17p*), unmutated immunoglobin heavy chain (*IGHV*) gene, and/or *TP53* mutations. Such patients are known to experience shorter time to progression, increased chemo-immunotherapy resistance, more aggressive disease, and poor prognoses^19–21^. The clinical implementation of an NGS assay to identify high-risk patients would allow clinicians to optimize treatment planning based on mutational profile. Timely guidance to inform initiation of BTKi versus venetoclax-based therapy is urgently needed^8,22–24^.

In 2021, the Canadian Agency for Drugs and Technologies in Health (CADTH) - now Canada's Drug Agency (CDA) - published a provisional funding algorithm for risk-based CLL treatment formulated on the basis of clinical evidence and in consultation with clinical experts^7^. The provisional funding algorithm was generated to help address implementation issues related to of previously evaluated treatments for adult patients with CLL. Following Health Canada approval and subsequent CADTH/CDA reimbursement recommendations, uncertainty remained regarding the appropriate prioritization of treatment across lines for this patient population. For this reason, the expert panel conducted a consensus-based approach alongside stakeholder consultation to recommend a provision algorithm involving the sequencing of the BTKi inhibitor, Acalabrutinib and Venetoclax based-therapy.

While cost-effectiveness considerations were explicitly considered in the individual reimbursement recommendations across treatments included in the provisional funding algorithm, the cost-effectiveness and budget impact of the algorithm is not known. This introduces additional uncertainty in payor decision-making, impeding healthcare system planning and implementation. The objective of this analysis is to investigate the cost-effectiveness of an NGS assay to sequence treatment initiation based on risk stratification for adult patients with CLL.

## Methods

### Model overview and structure

We used a Markov model to estimate the incremental costs and incremental effects of incorporating an NGS assay into a CLL treatment algorithm to stratify patients according to their risk status. The model structure was constructed using published CADTH/CDA algorithm and confirmed in consultation with oncologists and clinician scientists at BC Cancer, British Columbia, Canada^7^. We estimated costs and effects, in terms of quality-adjusted life-years (QALYs) over a 10-year time horizon, using a model cycle length of one month. The model included 5 mutually exclusive health states. Health states included in the model were “on treatment”, “disease progression,” “treatment-free interval”, “adverse events”, and “death.”

**Figure 1:**
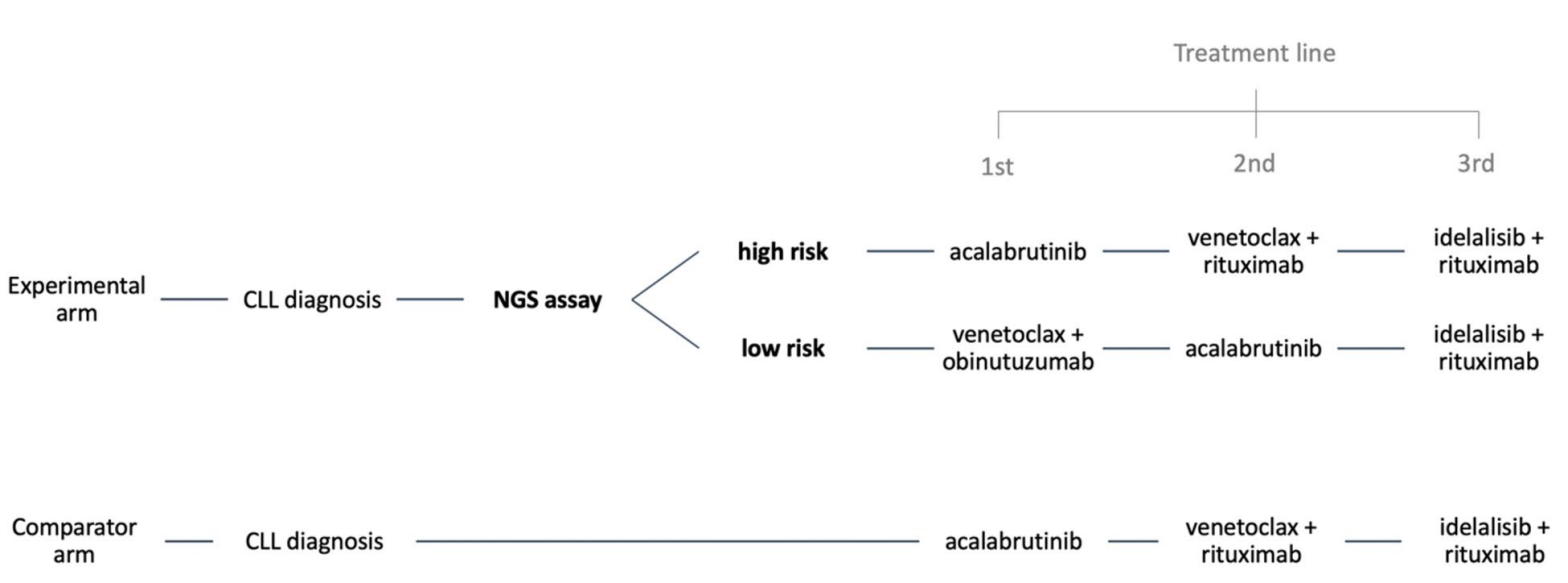
Conceptual model schematic. In the NGS assay strategy, the genetic test determines which patients are considered high and low-risk, and treatment is given accordingly. High risk patients initiate acalabrutinib, and low-risk patients initiate venetoclax + obinutuzumab. In the standard of care arm, all patients will receive first-line indefinite therapy with acalabrutinib.

Costs and effects were discounted as rate of 1.5%^25^. Our primary outcome for this analysis is the incremental cost-effectiveness ratio, defined as the incremental costs (**Δ**C) divided by incremental effectiveness (**Δ**E), at an assumed willingness-to-pay (WTP) threshold of $50,000 per quality adjusted life year gained. For base case results, we also report a net monetary benefit (NMB) statistics at a WTP threshold of $50,000 per quality adjusted life year gained, calculated as (ΔE × 50,000) – ΔC. Ninety-five percent confidence intervals are calculated as −/ + 1.96*standard deviation. We programmed the CEA in Python using the user-written EDAMAME (Economic Decision Analytic Markov Model Evaluation) package^26^. A schematic of our model is presented in Fig. 1.

The model includes two arms. At the outset of the model, all patients in the experimental (sequencing) arm underwent the hypothetical NGS assay which determined initial treatment selection, and correspondingly, subsequent treatments. For the purposes of this early-stage cost-effectiveness analysis, we assume no false positive or false negative results are reported. Patients stratified into the high-risk arm initiated acalaburtinib as first-line treatment, followed by venetoclax with rituximab as second-line therapy. Patients stratified as low-risk initiated venetoclax with obinutuzumab as first-line therapy, followed by acalabrutinib in second line. The comparator arm follows the model’s high-risk stratification treatment sequencing, beginning acalabrutinib as first-line treatment, followed by venetoclax with rituximab in second line. Across all model arms, idelalisib with rituximab was initiated for third-line treatment. Acalabrutinib was given to patients indefinitely until toxicity or disease progression, whereas the venetoclax and obinutuzumab regimen was given for 12 cycles.

### Model inputs

Transition probabilities for each treatment pathway were extracted from relevant clinical trials, as shown in Table 1^11,12,27–29^.

**Table 1:** Model parameters: clinical data used to generate model transition probabilities.

Model arm	Data	Clinical trial	Publication (if applicable)
First line			
Intervention arm only	Overall survival	ELEVATE-TN	Sharman et al.^12^
(BTKi—acalabrutinib monotherapy)	Progression free survival and adverse events	ELEVATE-TN	Sharman et al.^12^
Venetoclax + obinutuzumab	Overall survival, progression free survival and adverse events	CLL14	Al-Sawaf et al.^10^
Second line			
Intervention arm only	Overall survival	ELEVATE	Seymour et al.^29^
Venetoclax + rituximab	Progression free survival and adverse events	ELEVATE	Seymour et al.^29^
Acalabrutinib	Overall survival, progression free survival and adverse events	ASCEND	Ghia et al.^11^
Third line			
Both decision analytic model arms	Overall survival	ASCEND (control arm)	Ghia et al.^11^
Idelalisib + rituximab	Progression free survival and adverse events	ASCEND (control arm)	Ghia et al.^11^

Published Kaplan–Meier (KM) curves were digitized, extrapolated using parametric distributions, and converted into probabilistic time-dependent transition probabilities. In the reference case analysis, a Weibull distribution was used to extrapolate the data over the model time horizon. High-risk patients were defined as those that had *del17p* alteration, *TP53* mutation, and/or an unmutated *IGHV* status.

Survival estimates were sourced from relevant clinical trials depending on the line of therapy. Kaplan–Meier curves were digitized and extrapolated over the model time horizon. Utility values were derived from Beusterien et al*.*^30^ using the standard gamble technique to elicit utility values among patients with CLL. QALYs were calculated using these utility weights which were assigned to each mutually exclusive health state^30^. Costs of CLL treatment were sources from available Canadian list prices (see Table 2) and are presented in 2021 Canadian dollars.

**Table 2:** Model parameters: costs used in the economic model.

Parameter	Estimatea	Source
NGS assay	$1000	Clinician consultation (BC Cancer), based on test components
Acalabrutinib	$7615	List price/pERC report (no fixed duration)
Venetoclax + obinutuzumab	$10,400	List price/pERC report (average cost over 12 cycles)
Venetoclax + rituximab	$7614	List price/pERC report (average across 24 cycles)
Idelalisib + rituximab	$9754	List price/pERC report (no fixed duration)

^a^No measures of uncertainty were provided for treatment costs. As such, the analysis artificially included 25% variability in either direction for all costs associated with the model with the exception of the genetic test which was allowed to vary 75% owing to its stage of development. Costs for treatments are reported as average per cycle costs.

Additional costs for adverse events were obtained from the published literature. Specific parameter values for each health state are presented in Table 3.

**Table 3:** Model parameters: utility values used to calculate quality-adjusted life-years (QALYs).

Utilities	Mean	SD	Source
On first-line treatment	0.78	0.14	Beusterien et al.^30^
On second-line treatment	0.71	0.17	Beusterien et al.^30^
On third-line treatment	0.65	0.22	Beusterien et al.^30^
Adverse event (major: 3/4 resulting in treatment discontinuation)	0.69	0.18	Beusterien et al.^30^
Adverse event (minor: 1/2 continue with treatment)	0.73	0.17	Beusterien et al.^30^
Treatment-free interval	0.91	0.11	Beusterien et al.^30^
Relapse	0.68	0.2	Beusterien et al.^30^

Our model assumes that the NGS assay would accurately detect 50% of patients as high-risk, at a cost of $1000 CAD. The basis for the proportion of high-risk patients is based on available evidence and expert clinical consultation^21^. Assumed cost of the NGS assay is informed by a conservative estimate of the individual costs for detecting mutations that would categorize a patient as high-risk.

### Analysis

We conducted a probabilistic analysis in alignment with guidance for economic evaluation published by CADTH/CDA^25^. Whenever possible, transition probabilities were time-dependent over the model time horizon. For the probabilistic analysis, 1000 iterations were run and plotted on a cost-effectiveness plane. We conducted probabilistic scenario analyses by varying the predictive probabilities of the NGS assay and an additional analysis varied the cost of the NGS assay. The rationale underlying the analytic approach is that the value of this NGS assay is linked to the prevalence of, and ability to, detect high-risk patients. If all patients are high-risk or the genetic test identifies all patients as high-risk, this will almost exactly match the standard of care arm where all patients are treated with acalabrutinib in the first-line setting.

### Ethics and consent to participate

This study was approved by the University of British Columbia BC Cancer Behavioural Research Ethics Board (H18-00490). No human subjects were used as part of this research.

## Results

Results of our analysis illustrate that the treatment algorithm incorporating the NGS assay was associated with both greater costs and effects. The intervention of a genetic assay into a treatment algorithm for patients with CLL resulted in a cost of $155,276 relative to the standard of care cost of $137,130 in the absence of risk stratification, with an incremental cost of $18,146 (95%CI − 29,398.82, 69,032.13); see Table 4).

**Table 4:** Results in terms of incremental costs, incremental effects (quality-adjusted life-years) from the reference case probabilistic analysis.

Model arm	Cost (95% CI*)	Δ Costs	QALYs (95% CI)	Δ QALYs	ICER	NMB
Genetic test arm	$155,276 (133,359.3, 177,192.7)	$18,146 (95%CI -29,398.82, 69,032.13)	2.05 (1.462, 2.638)	1.01 (95%CI 0.12, 1.91)	$18,040 (95% CI $16,491-$19,501)	$32,354 (95% CI 30,804.99–33,815.99
Comparator arm	$ 137,130 (92,142.12, 182,117.90)		1.04 (0.3736, 1.7064)			

Δ: Incremental.

**Figure 2:**
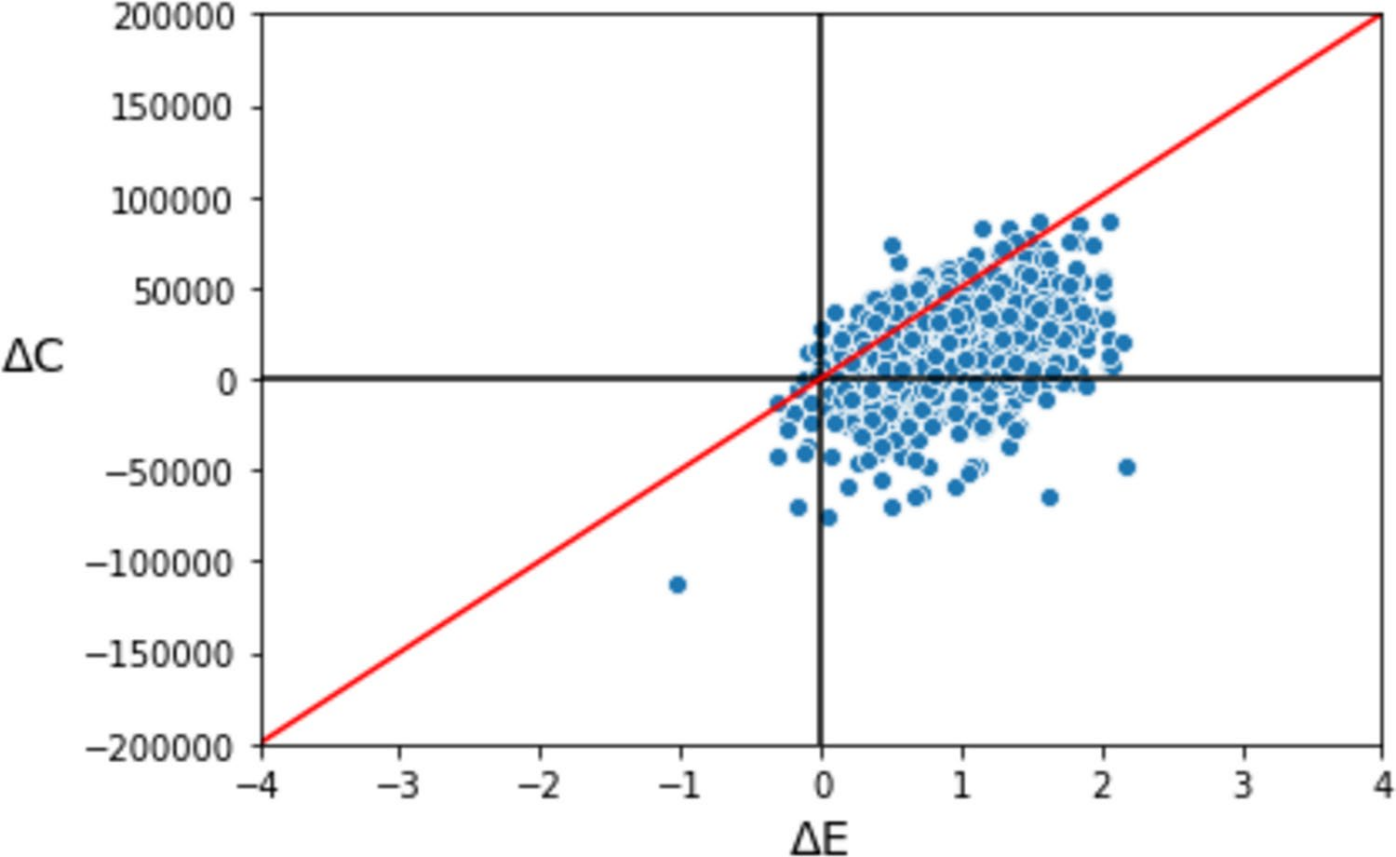
Scatterplot of incremental costs and incremental effects (QALYs) based on 1000 probabilistic model iterations. Dots/simulations to the right of the vertical axis represent a scenario where the intervention arm is considered more effective to the standard of care. Similarly, dots/simulations below the horizontal axis represent simulations where the intervention arm was estimated to be less costly than the standard of care.

The NGS assay intervention also resulted in an incremental gain in QALYs of 1.01 (95%CI 0.12, 1.91). The resulting ICER was $18,040 (95% CI $16,491-$19,501) per QALY gained (see Fig. 2). Using a WTP of $50,000 per QALY gained, the corresponding net monetary benefit statistic was $32,354 (95% CI 30,804.99 – 33,815.99), indicating a cost-effective intervention.

**Figure 3:**
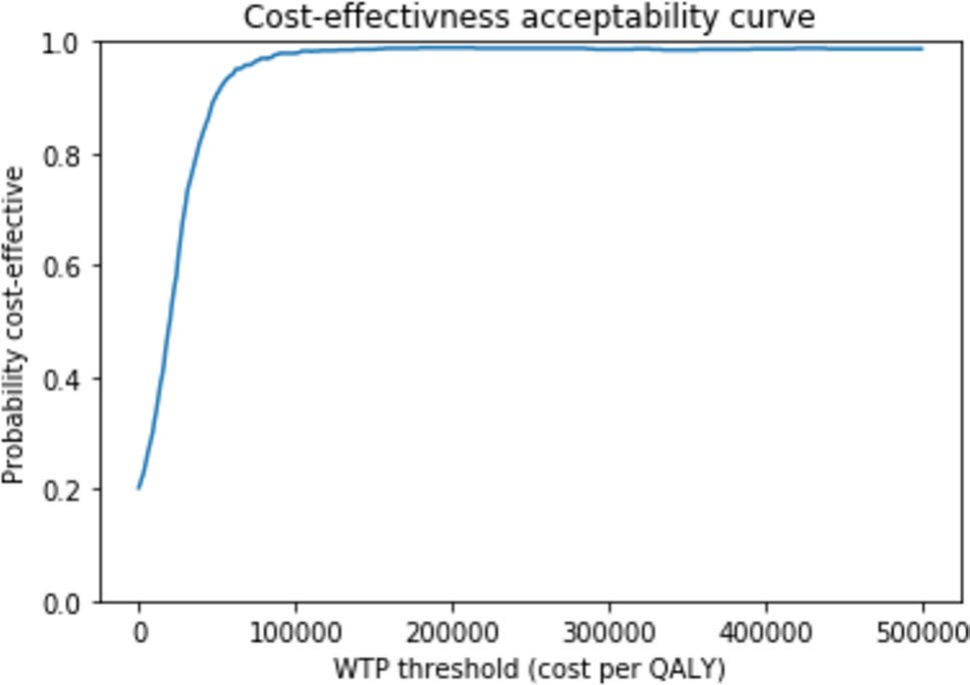
Cost-effectiveness acceptability curve which represents the probability that the NGS assay intervention would be considered cost-effective at various thresholds of WTP per quality-adjusted life-year (QALY) gained.

At a willingness-to-pay (WTP) threshold of $50,000 per QALY gained, the probability of the introduction of the NGS assay being cost-effective was 80%. Approximately 19% of the 1000 probabilistic iterations resulted in a scenario where the addition of the genetic test into the algorithm resulted in lower costs and greater effects (QALYs) (i.e., dominated the standard of care; see Fig. 3).

In scenario analyses, the assumed cost of the NGS assay varied from $0 to $2000. The resulting ICER per QALY gained varied from $17,361 to $19,034 per QALY gained, respectively. This analysis demonstrates that while the NGS assay cost contributes to the ICER, the cost associated with treatment far outweigh cost of testing. An additional probabilistic sensitivity analysis explored the proportion of patients who would be identified as high-risk. If 100% of patients were stratified as high-risk, the sequence of treatments would be almost identical to the comparator arm of the model and the resulting dominant ICER of $ − 628 (95% CI − $35,585 to $17,669). If the NGS assay stratified 75% of patients at low-risk, the introduction of the assay would result in an ICER of $18,560 per QALY gained (Table 5).

**Table 5:** Scenario analyses of varying the cost of the NGS assay and the probability of accurately predicting high-risk patients. All analyses are probabilistic.

Percent high risk	Δ Costs	Δ Effects	ICER
0%	$36,347	1.92	$18,892
25%	$27,247	1.46	$18,560
50% (reference case)	$18,146	1.01	$18,040
75%	$9,045	0.55	$16,541
100%	$− 55.13	0.09	$− 628

Cost of NGS assay	Δ Costs	Δ Effects	ICER
0	$17,535	1.01	$17,361
500	$17,655	1.01	$17,542
1000 (reference case)	$18,146	1.01	$18,040
1500	$18,646	1.01	$18,537
2000	$19,146	1.01	$19,034

## Discussion

Within publicly funded healthcare systems, genetic testing and treatment costs pose budgetary and affordability challenges, particularly for more prevalent cancers^31^. Optimizing treatment sequencing remains a policy priority to ensure that treatments are provided to the right patients at the right time. This analysis evaluated the introduction of an NGS assay to determine risk status among adult patients with CLL initiating front-line treatment. The CADTH/CDA treatment algorithm provided a basis for treatment of patients with CLL, with cost-effectiveness of the algorithm yet to be established. The hypothetical NGS assay used in our analysis would allow clinicians to sequence treatments for patients with CLL according to their risk status, reserving more costly treatments for later therapy lines. Results of this evaluation suggest that an NGS assay to risk-stratify patients with CLL could be cost-effective at commonly cited thresholds of WTP per QALY gained. This analysis demonstrates that CADTH/CDA’s algorithm that delivers treatment according to risk based on the status of *del17p*, *TP53* mutation, and *IGHV* unmutated status is likely cost-effective from the perspective of a Canadian provincial healthcare system. Notably, reserving costly, long-term treatment strategies such as acalabrutinib for patients requiring second line treatment may be a cost-effective treatment sequencing approach to further improve outcomes for patients identified as low-risk.

The ability to accurately predict risk status in patients with CLL would provide value to both patients and reimbursement decision-makers. Recent studies have shown that incorporating a genetic test to determine cell of origin in diffuse large B-cell lymphoma to personalize treatment incurred survival benefit and was potentially cost-effective^32,33^. Emerging clinical validation studies have shown that patient risk stratification can help identify patients with classical Hodgkin lymphoma likely to experience relapse after stem cell transplant and would therefore benefit from consolidation therapy^34^. Our study adds to the evidence base for the potential value of risk stratification tests via NGS assays to enhance efficient delivery of healthcare services and improve patient health outcomes. To ensure that our analysis is responsive to an evolving evidence base and able to support decision-making, we conducted extensive scenario analyses.

Evidence is emerging rapidly to inform clinical and cost effectiveness of CLL treatment strategies^13,17,18,35–39^. Munir 2020 conducted a cost-effectiveness analysis of acalabrutinib monotherapy compared to chlorambucil-obinutuzumab, among previously untreated CLL patients using patient-level survival data sourced from the ELEVATE-TN trial^17^. Treatment sequencing used by Munir et al. differ from the current analysis, wherein patients who experienced disease progression after chlorambucil-obinutuzumab treatment proceeded to ibrutinib, while patients progressing on first-line acalabrutinib received rituximab with venetoclax or bendamustine. Applying a lifetime horizon of 30 years, investigators found that acalabrutinib monotherapy resulted in an ICER of £30 701 per QALY. While acalabrutinib treatment resulted in higher treatment costs than chlorambucil-obinutuzumab due to extended treatment duration, costs were partially offset by lower subsequent treatment costs and fewer adverse events. Consistent with our findings, key cost drivers were proportion of patients receiving post-progression ibrutinib or venetoclax and BTKi price.

Our work builds on evidence informing the cost-effectiveness of treatment sequencing strategies for CLL, namely to inform genomics-guided sequencing of acalabrutinib, from the perspective of a Canadian provincial health system. Results highlight several avenues for further research. Firstly, our NGS assay is based on a hypothetical test. Evidence informing the effectiveness and cost-effectiveness of such tests in identifying optimal treatment candidates is required, either prior to or alongside implementation or re-evaluation. Further, an accurate characterization of test characteristics (e.g. sensitivity, specificity, and cost) and real-world uptake would enhance the validity of input parameter estimates and reduce both evidentiary and decision uncertainty.

### Limitations

This analysis should be interpreted in light of limitations. The cost-effectiveness analysis relied on published literature, with assumptions validated through clinical expert consultation. For this reason, our analysis is limited by the limitations of the referenced clinical trials, which include short follow up periods, highly selected patient participants, and small sample sizes. In addition, we did not conduct a formal systematic review to inform this model. Rather, in consultation with treating hematologist-oncologists at BC Cancer, the most timely and appropriate data sources were identified and determined as appropriate for this early-stage model. As additional data or evidence emerges, our model can be updated as appropriate, to ensure relevance to this patient population and healthcare system setting. Treatment costs and health state utility values used to calculate QALYs were also sourced from published literature. The costs are likely to be an overestimate of the true costs paid by a Canadian health system. Utilities were taken from a single study and elicited using the standard gamble technique which avoids using multiple sources and does not require an algorithm to weight responses to a questionnaire, as is common. Finally, for the purposes of this early-stage cost-effectiveness analysis, we assumed that the hypothetical assay perfectly assigns patients into high and low-risk categories, which may not reflect real world testing strategies. Future economic evaluations of risk stratification assays will be strengthened by integration of assay validity, such as false positive and negative rates, and predictive value.

Our study assumed that the NGS assay would stratify 50% of patients as high-risk. The choice of this value was informed by published evidence and clinical expert consultation. This assumed value may change as further research is conducted to identify genetic abnormalities that determine risk among CLL patients. To appreciate variability of this parameter, scenario analyses were conducted that altered this specific value. This technology is likely to evolve as further research is conducted and scenario analyses are sufficiently flexible to remain useful to decision-makers if the specific characteristics change over time. Regarding treatment cost, while we acknowledge that the list price is seldom the actual price paid in a particular jurisdiction, using list prices represent a conservative estimate of costs in each treatment arm. Finally, our study did not incorporate broader considerations of what could be important in decision-making about new health technologies such as equity and climate impact.

## Conclusion

Investigating the cost-effectiveness of recommended treatment algorithms is critical to ensure affordability and appropriate treatment access. As evidenced by this work, the use of an NGS assay to stratify CLL patients as high-risk to inform treatment sequencing is potentially cost-effective. Robust evidence able to further reduce decision uncertainty will require the integration of real-world evidence informing NGS-assay accuracy, cost, adoption and uptake in clinical practice.

## Data Availability

All data generated or analysed during this study are included in this published article.
